# Supplementary material for: Effect of mosquito saliva from distinct species on human dermal endothelial cell function in vitro and West Nile virus pathogenesis in vivo
Source: Emerg Microbes Infect. 2025 Jun 20;14(1):2502006. doi: 10.1080/22221751.2025.2502006 (PMC12312492; doi:10.1080/22221751.2025.2502006)
Supplement: Supplementary_Visser_revised_clean.docx [file TEMI_A_2502006_SM1029.docx]

**Supplementary**


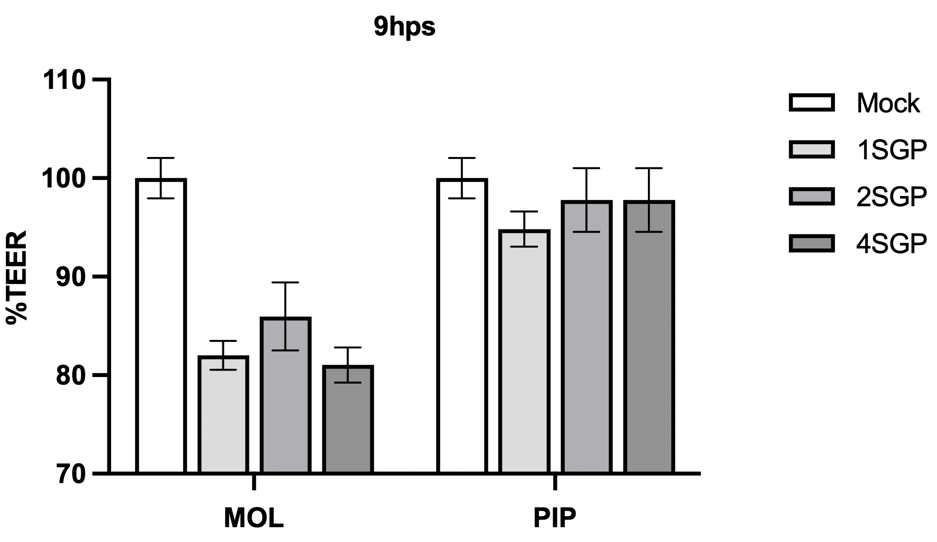


**Supplementary Figure 1. Effect of increased amount of salivary gland pairs (SGPs) on the trans-endothelial electrical resistance (TEER).** An equivalent of either 1, 2, or 4 Cx. pipiens molestus (MOL) (1.95μg, 3.90μg, and 7.80μg total protein, respectively) or Cx. pipiens pipiens (PIP) (1.98μg, 3.96μg, and 7.92μg total protein, respectively) SGP was added to primary human dermal microvascular endothelial cells after which TEER was measured at 9 hours post-stimulation (hps). Data from one individual experiment.

**Supplementary Table 1.** Top 5 most differentially present peptides found in Cx. pipiens molestus versus Cx. pipiens pipiens salivary gland extract in mass spec pilot analysis.

| Peptide sequences | Protein Accession | Presence *molestus* | Presence *pipiens* | Putative protein |
| --- | --- | --- | --- | --- |
| DVTFSDYIRPICLPLSDELR;FDRDVTFSDYIRPICLPLSDELR;NIPDVYTNVASYVDWIESK | A0A8D8GZ17;A0A8D8JX55 | High | Undetected | CLIP domain-containing serine protease |
| YVLTGASCVFR | A0A8D8F1U3 | High | Undetected | CLIP domain-containing serine protease |
| RSPNELQESQLCALGR;SPNELQESQLCALGR | A0A8D7ZVM8;A0A8D7ZWQ4;A0A8D8DVJ7 | High | Undetected | Serine protease persephone-like/DESC4/Hayan-like |
| TNAPGICRPVAECPSVIADIR | A0A8D8AYL9;A0A8D8KAB8 | High | Undetected | Venom protease-like isoform |
| LAEPVATSSWVRPVCLPER;QVAGVGVVHELAGILNHMK | A0A8D8AXX0;A0A8D8DFT6;A0A8D8DHD4;A0A8D8GL83 | High | Low | Serine protease |
